# Analysis of the In Vivo and In Vitro Effects of Photodynamic Therapy on Breast Cancer by Using a Sensitizer, Sinoporphyrin Sodium: Erratum

**DOI:** 10.7150/thno.62096

**Published:** 2021-05-15

**Authors:** Xiaobing Wang, Jianmin Hu, Pan Wang, Shaoliang Zhang, Yichen Liu, Wenli Xiong, Quanhong Liu

**Affiliations:** 1 Key Laboratory of Medicinal Resources and Natural Pharmaceutical Chemistry, Ministry of Education, National Engineering Laboratory for Resource Developing of Endangered Chinese Crude Drugs in Northwest of China, College of Life Sciences, Shaanxi Normal University, Xi'an, Shaanxi, People's Republic of China.; 2 Qinglong High-Tech Co., Ltd, Yichun, Jiangxi, People's Republic of China

We noticed two errors in the initially published version of this article about the DVDMS alone group in transwell (Fig. 5B) and H&E (Fig. 9 upper part) assays. The correct representative Figures are as follows:

The corrections made in this erratum do not affect the original conclusions. The authors apologize for any inconvenience or misunderstanding that this error may have caused.

## Figures and Tables

**Fig 5:**
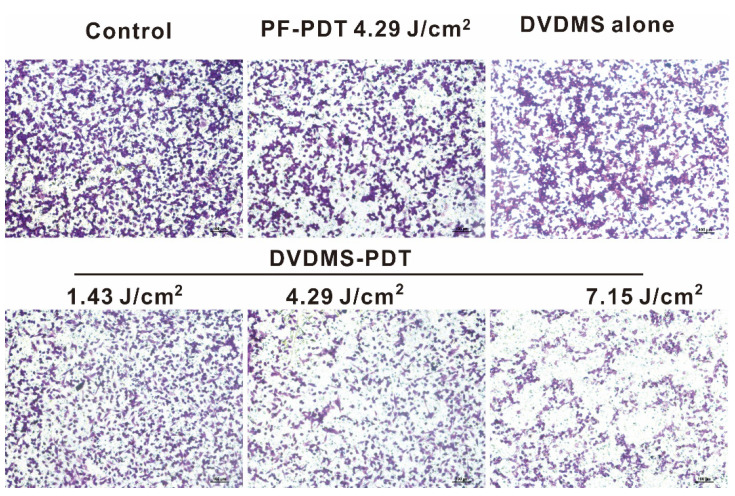
**B.** Analyses of cell migration using a transwell assay.

**Fig 9:**
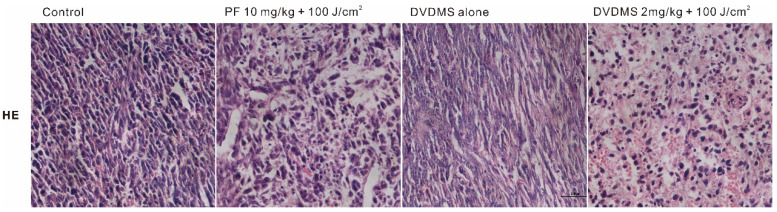
Tumor sections were stained with hematoxylin and eosin (HE).
